# HIF-1alpha Expression Profile in Intratumoral and Peritumoral Inflammatory Cells as a Prognostic Marker for Squamous Cell Carcinoma of the Oral Cavity

**DOI:** 10.1371/journal.pone.0084923

**Published:** 2014-01-09

**Authors:** Suzanny Oliveira Mendes, Marcelo dos Santos, Gabriela Tonini Peterle, Lucas de Lima Maia, Elaine Stur, Lidiane Pignaton Agostini, Marcos Brasilino de Carvalho, Eloiza Helena Tajara, Iúri Drumond Louro, Leonardo Oliveira Trivilin, Adriana Madeira Álvares da Silva-Conforti

**Affiliations:** 1 Programa de Pós Graduação em Biotecnologia - Renorbio, Universidade Federal do Espírito Santo, Vitória, Espírito Santo, Brazil; 2 Departamento de Medicina, Universidade Federal do Rio Grande do Norte, Caicó, RN, Brazil; 3 Laboratório de Biologia Molecular, Hospital Heliópolis, São Paulo, São Paulo, Brazil; 4 Departamento de Biologia Molecular, Faculdade de Medicina de São José do Rio Preto, São José do Rio Preto, São Paulo, Brazil; Sapporo Medical University, Japan

## Abstract

The HIF-1 transcriptional complex is responsible for controlling transcription of over 100 genes involved in cell hypoxia response. HIF-1alpha subunit is stabilized in hypoxia conditions, creating the HIF-1 nuclear transcription factor. In inflammatory cells, high HIF-1alpha expression induces lymphocytic immunosuppression, decreasing tumoral antigen recognition, which promotes tumor growth. The present work investigated the relationship between HIF-1alpha expression in lymphocytes populating the intratumoral and peritumoral region of 56 patients with oral cancer. Our data indicates a prognostic value for this expression. High HIF-1alpha expression in peritumoral inflammatory cells is significantly related to worse patient outcome, whereas high expression in the intratumoral lymphoid cells correlates with a better prognosis. A risk profile indicating the chance of disease relapse and death was designed based on HIF-1alpha expression in tumoral inflammatory cells, defining low, intermediate and high risks. This risk profile was able to determine that high HIF-1alpha expression in peritumoral cells correlates with worse prognosis, independently of intratumoral expression. Low HIF-1alpha in tumor margins and high expression in the tumor was considered a low risk profile, showing no cases of disease relapse and disease related death. Intermediate risk was associated with low expression in tumor and tumor margins. Our results suggest that HIF-1alpha expression in tumor and peritumoral inflammatory cells may play an important role as prognostic tumor marker.

## Introduction

Head and neck cancer is a significant cause of mortality and morbidity worldwide, presenting approximately 600,000 new cases yearly [1], reaching the sixth leading cause of death by cancer [2]. Whereas tumors of the oral cavity contribute with 389,000 new cases per year, with a mortality rate of 50% [3]. Squamous cell carcinoma is the most common histological variant, making for 90% of all head and neck tumors [4].

Tumor histopathological results have been considered supplementary prognostic data, helping in the therapeutic decision making [5]. Among these results, positive tumor margins have been predictive of disease relapse, frequently influencing the therapeutic decision [6]. Another important information resulting from the histopathological analysis is the presence of inflammatory cells in the intratumoral region, however the role of this infiltrate is still controversial.

In 1863, Rudolph Virchow described for the first time the presence of inflammatory cells in the neoplasic tissue [7]. Since then, inflammation has been associated with inhibition or induction of tumor growth [8]. However, tumor inflammatory cells are generally living in hypoxic conditions [9], which induces expression of HIF-1alpha protein (Hypoxia Induced Factor-1 alpha), a partner of HIF-1beta in the HIF-1 heterodimer, resulting in the major cellular response to hypoxia [10]. This complex triggers specific mechanisms to avoid cell death caused by hypoxia, as well as causes numerous microenvironment changes through the transcription of over 100 genes involved in cell metabolism, angiogenesis, glycolysis and other intracellular processes [11]. Therefore, HIF-1alpha expression has been studied in solid tumors such as breast [12] colorectal [13] and head and neck tumors [14].

Because tumor inflammatory cells are generally living under hypoxic conditions and because HIF-1alpha can interact with other inflammatory protein complexes, it plays a fundamental role in the regulation of tumor inflammation [9], [15]–[16].

The present study aims to determine the role HIF-1alpha expression in specific places of the tumoral microenvironment as a prognostic marker in tumors of the oral cavity.

## Materials and Methods

### Ethics

This study was approved by the Research Ethics Committee of the Heliópolis Hospital on 06/10/2008 (CEP n° 619) and an informed consent was obtained from all patients enrolled.

### Samples

Samples were collected by the Head and Neck Genome Project (GENCAPO), a collaborative consortium created in 2002 with more than 50 researchers from 9 institutions in São Paulo State, Brazil, which aim is to develop clinical, genetic and epidemiological data of HNSCC. In this study, 56 tumor and 44 nontumor surgical margins were obtained and used for immunohistochemical analysis of HIF-1alpha expression in lymphoid cells of patients with oral squamous cell carcinoma, surgically treated at the Head and Neck Surgery Department of the Heliópolis Hospital, São Paulo, Brazil, during the period of January/2002 to December/2008. Clinical follow-up was at least 48 months after surgery. Previous surgical treatment, distant metastasis, no removal of cervical lymph nodes and positive surgical margins were exclusion criteria. Histopathological slides were reviewed by a senior pathologist to confirm the diagnosis and select appropriate areas for immunohistochemical analysis. Tumors were classified according to the TNM system (3^rd^ edition) [17].

Patient gender and age characterization showed age varying from 34–81 years, with a mean of 56 years (df±11 years), being 48 (85.7%) males and 8 (14.3%) females. Anatomical tumor location was distributed as follows, 22 (39.3%) were on the tongue, 10 (17.9%) on inferior gums, 20 (35.7%) on the floor of the mouth and 4 (7.1%) on the retromolar area. Clinical and pathological tumor characteristics are described in Table 1.

**Table 1:** Clinical and pathological features and their relation with HIF-1alpha expression in lymphoid cells of the inflammatory infiltrate; intratumoral and peritumoral.

Clinical and pathological features	HIF-1alpha expression in lymphoid cells of inflammatory infiltrate													
	Peritumoral							Intratumoral						
	Total		Weak		Strong		*p*	Total		Weak		Strong		*p*
	No.	(%)	No.	(%)	No.	(%)		No.	(%)	No.	(%)	No.	(%)	
**Tumor stage**														
I	2	4.5	2	8.3	0	0.0	0.175	2	3.6	1	2.3	1	7.7	0.656
II	11	25.0	8	33.4	3	15.0		12	21.4	10	23.3	2	15.4	
III	9	20.5	3	12.5	6	30.0		13	23.2	9	20.9	4	30.8	
IV	22	50.0	11	45.8	11	55.0		29	51.8	23	53.5	6	46.2	
**Tumor size (T)** a														
T1+T2	21	47.7	12	50.0	9	45.0	0.529	24	42.9	16	37.2	8	61.5	0.290
T3	6	13.6	2	8.3	4	20.0		11	19.6	9	20.9	2	15.4	
T4	17	38.6	10	41.7	7	35.0		21	37.5	18	41.9	3	23.1	
**Lymph-nodes (N)** a														
Absent	22	50.0	14	58.3	8	40.0	0.226	24	42.9	21	48.8	3	23.1	0.091
Present	22	50.0	10	41.7	12	60.0		32	57.1	22	51.2	10	76.9	
**Diferentiation grade**														
Well	19	43.2	10	41.7	9	45.0	0.942	24	42.9	21	48.8	3	23.1	0.074
Moderate	21	47.7	12	50.0	9	45.0		28	50.0	18	41.9	10	76.9	
Poor	4	9.1	2	8.3	2	10.0		4	7.1	4	9.3	0	0.0	
**Relapse**														
No	21	47.7	15	68.2	6	30.0	0.013	27	48.2	17	43.6	10	76.9	0.037
Yes	21	47.7	7	31.8	14	70.0		25	44.6	22	56.4	3	23.1	
Not available^b^	2	4.5						4	7.1					
**Death**														
No	24	54.5	17	73.9	7	36.8	0.016	31	55.4	21	52.5	10	76.9	0.108
Yes	18	40.9	6	26.1	12	63.2		22	39.3	19	47.5	3	23.1	
Not available^b^	2	4.5						3	5.4					
**Total**	**44**	**100.0**	**24**	**(54.5)**	**20**	**(45.5)**		**56**	**100**	**43**	**(76.8)**	**13**	**(23.2)**	

^a^ TNM classification (3rd edition) [17].

^b^ Not available – Not considered in statistical calculations.

### Immunohistochemistry

Anti- HIF-1alpha polyclonal antibody (Millipore Corporation, USA) was used in the IHC reaction at a 1∶150 dilution. Positive (breast cancer controls) and negative (absence of primary antibody) controls were used. Sample scoring was performed by semiquantitative microscopic analysis, considering the number of stained cells and signal intensity. Considering the percentage of HIF-1alpha immune-positive lymphoid cells, a score of 0 was given when all cells negative; a score of 1 when 1–25% of cells were positive, 2 when 25–50% of cells were positive and 3 when >50% of cells were positive. Sigma intensity was scored as negative (0), weak (1), moderate (2) and strong (3). Both scores were multiplied [18]–[19] and the resulting score was used to categorize HIF-1alpha expression as weak (≤3) and strong (>3).

### Statistical Analysis

Chi square and Fisher exact tests were used for association analysis and confirmation was obtained by the Lilliefors test (significance considered when p<0.05). Multivariate logistic regression was used to obtain odds ratio (OR) and confidence intervals (CI 95%). Survival was calculated by the number of months between surgery and death for each patient or the last appointment in case the patient was alive. In order to calculate disease-free survival, endpoint was the date of disease relapse. The Kaplan-Meier model was used for survival analysis, using the Wilcoxon p-value and the Cox Proportional Hazards to adjust p-values and obtain hazard ratio (HR). Statistical calculations were performed using the Epi Info® v3.4.3, 2007 and Statsoft Statistica® v7.0.61.0 softwares.

## Results

### HIF-1alpha expression in peritumoral inflammatory infiltrate

**Figure 1:**
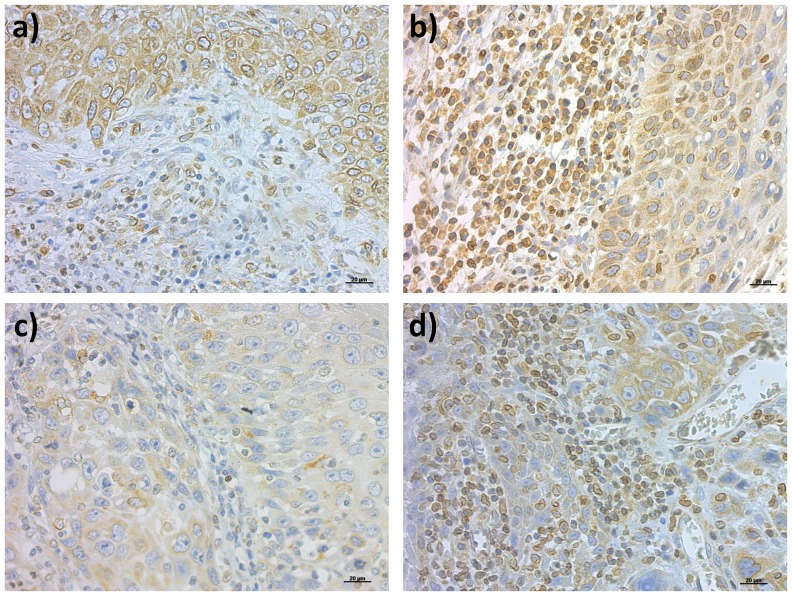
Immunohistochemistry. **a and b.** Weak and strong HIF-1alpha expression in peritumoral inflammatory infiltrates; **c. and d.** Weak and strong HIF-1alpha expression in intratumoral inflammatory infiltrates. Magnification was 400×.

HIF-1alpha expression was studied in 44 samples of peritumoral inflammatory infiltrates, from which strong expression was detected in 24 (54.5%) samples and weak expression in 20 (45.5%, Figure 1a and 1b). HIF-1alpha expression was not statistically related with tumor stage (p = 0.175), tumor size (p = 0.529), node positivity (p = 0.226) or differentiation grade (p = 0.942) (Table 1).

HIF-1alpha expression was significantly associated with disease relapse and death (p = 0.013 e p = 0.016, respectively). Multivariate analysis revealed that strong HIF-alpha expression is a marker of disease relapse and disease related death (OR = 5.75, CI = 1.03–32.06 e OR = 5.63, CI = 1.09–28.98, respectively), increasing their risk over 5 times, when compared with weak expression.

**Figure 2:**
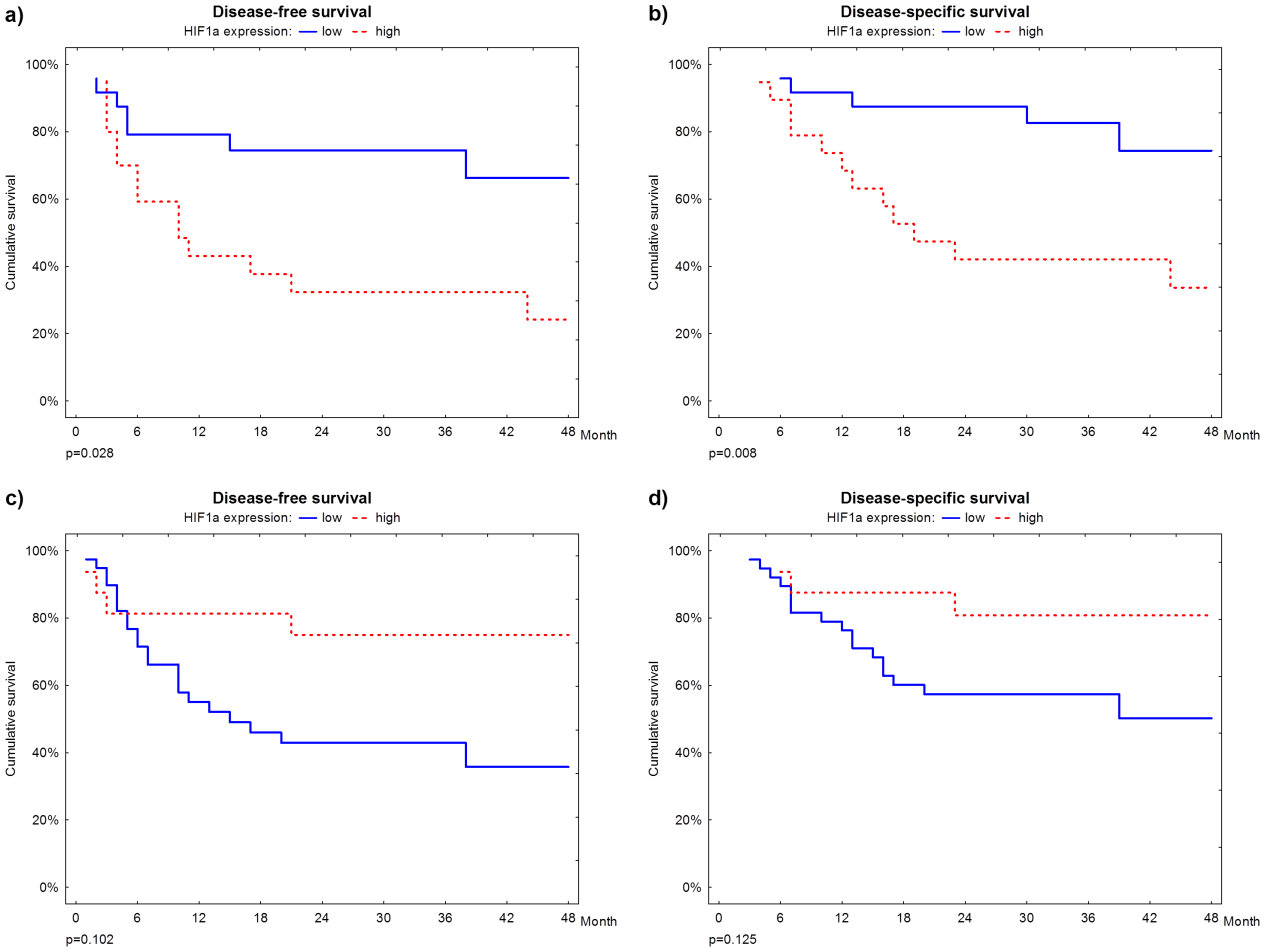
Survival plots. **a. and b.** Disease-free and disease-specific survivals according to HIF-1alpha expression in lymphoid cells of the peritumoral infiltrate; **c. and d.**: Disease-free and disease-specific survivals according to HIF-1alpha expression in lymphoid cells of the intratumoral infiltrate.

High HIF-1alpha expression in lymphoid peritumoral cells was correlated with worse disease-free and disease-specific survivals (p = 0.028 e p = 0.008, respectively). During the 24 month after surgery follow up, approximately 20% of patients with weak HIF-1alpha expression presented disease relapse and 10% died, whereas in the same period, 70% of patients with strong expression relapsed and 60% died of disease related causes (Figure 2a and 2b). Multivariate analysis showed that strong HIF-alpha expression is an independent risk factor for worse disease-free and disease-specific survival (HR = 3.29; CI = 1.10–9.86 e HR = 3.88; CI = 1.24–12.07, respectively), augmenting both risks over 3 times, when compared with weak expression.

### HIF-1alpha expression in the intratumoral inflammatory infiltrate

HIF-1alpha expression was studied in 56 samples of intratumoral lymphocytic inflammatory cells, in which 43 (76.8%) samples showed weak and 13 (23.2%, Figure 1c and 1d) showed strong expression. HIF-1alpha was not statistically associated with tumor stage (0.656), tumor size (p = 0.290), node positivity (p = 0.091) or differentiation grade (p = 0.074, Table 1).

HIF-1alpha did not correlate with death, but was significantly related with disease relapse (p = 0.037, Table 1). However, multivariate analysis did not confirm HIF-1alpha as an independent marker of disease relapse (OR = 9.73, CI = 0.82–115.25). In addition, HIF-1alpha expression was not related with disease-free or disease-specific survival (p = 0.102 and p = 0.125, respectively, Figure 2c e 2d).

### HIF-1alpha lymphocytic profile

In order to better test the impact of HIF-1alpha expression in inflammatory infiltrates and its correlation with patient prognosis, we have established a HIF-1alpha expression profile, according to the localization of the infiltrate in relation to the tumor, allowing us to determine 3 risk profiles, namely: low, intermediate and high risk profiles, as described in table 2.

**Table 2:** HIF-1alpha risk profile and correlation with disease relapse and death.

HIF-1alpha risk profile			Relapse					Death				
			No		Yes		*p*	No		Yes		*p*
Risk	Peritumoral	Intratumoral	No.	(%)	No.	(%)		No.	(%)	No.	(%)	
Low	Weak	Strong	5	26.3	0	0.0	0.027	5	22.7	0	0.0	0.014
Intermediate	Weak	Weak	9	47.4	6	33.3		11	50.0	5	33.3	
High	Strong	Strong or Weak	5	26.3	12	66.7		6	27.3	10	66.7	

This risk profile was significantly associated with disease relapse and disease-related death (p = 0.027 e 0.014, respectively). No patient classified as low risk presented disease relapse or died due to the disease, whereas approximately 70% of high risk patients showed disease relapse or died due to disease-related causes (Table 2).

**Figure 3:**
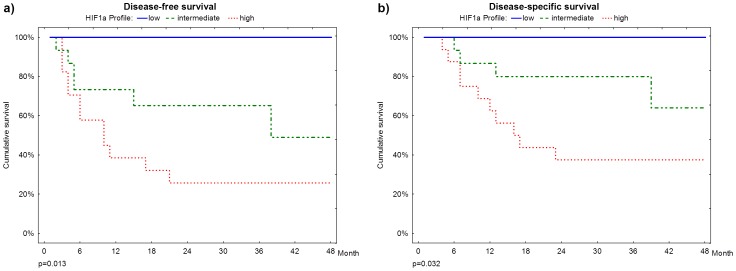
Survival plots. **a. and b.** Disease-free and disease-specific survivals according to HIF-1alpha risk profile.

Moreover, HIF-1alpha risk profile was statistically related with disease-free survival and disease-specific survival (p = 0.013 e p = 0.032, respectively). During the 24 month after surgery follow up, no patient classified as low risk died or relapsed, whereas approximately 70% of high risk patients relapsed and 60% died of disease-related causes (Figure 3a and 3b).

### Discussion and Conclusions

A major role of the immune system is immunologic surveillance, which keeps transformed cells in constant check, attempting to destroy them before they become full blown tumors. Tumoral inflammatory infiltrates, present in several cancer types, have a clinical significance better understood in breast and colorectal tumors [20,−21], being interpreted by the pathologist as prognostic predictive factors. In squamous cell carcinoma of the oral cavity, this infiltrate is evaluated by the pathologist, but there is yet no prognosis prediction based on it.

The vast majority of research studies try to understand HIF-1alpha expression in tumor cells and its prognosis significance [12], [14], [22] and to our knowledge this is the first evaluation of HIF-1alpha expression in peritumoral or intratumoral inflammatory cells in humans. This might be of particular importance due to its role in the tumor microenvironment and lymphocytic suppression [15]–[16].

Based on our results, we have concluded that high HIF-1alpha expression is related with the worst possible prognosis, including disease relapse and disease-related death. Patients with high HIF-1alpha expression had a 5 times higher risk of relapse and death, when compared with low expression. Moreover, strong expression was an independent risk factor for disease-free and disease-specific survivals, showing a 3 times increased risk.

Our data is supported by de Thiel et al., who showed that lymphoid cells might not work appropriately when expressing high HIF1-alpha [15]. Our hypothesis is that high expression may inhibit lymphoid cells in the peritumoral infiltrate, due to the interaction between cytokines and HIF-1alpha, resulting in less tumor antigen recognition by lymphocytes, as well as anti-inflammatory signals and less tumor attack [16].

In conclusion, it seems reasonable that peritumoral infiltrate cells have a unique role in the anti-tumor response, which can be observed in the risk profile analysis, as weak expression in the tumor margins and strong expression inside the tumor correlate with a low risk profile and better prognosis, with no disease relapse or death. Weak expression in tumor margins and weak expression inside the tumor increased relapse and death risk by 30%, defining an intermediate risk, as compared to strong expression in tumor margins, which defines a high risk independent of any other factor.

We believe the observed difference in expression and clinical consequence is due to the diverse microenvironment nature. Inside the tumor, a hypoxic microenvironment stabilizes HIF-1alpha, which rapidly induced expression of VEGF, promoting angiogenesis [11], therefore increasing tumor vascularization and oxygenation, a response that may lead to a better radiotherapic treatment efficacy [14]. Furthermore, higher HIF-1alpha expression inside the tumor may induce a pro-inflammatory cascade responsible for tumor combat through TNF receptor activation [23], hence determining a better prognosis.

Our results suggest that evaluation of HIF-1alpha expression in the inflammatory infiltrate may be used as a prognosis tumor marker for squamous cell carcinoma of the oral cavity.
